# Motor and cognitive function after unipolar depressive episodes: a 6 years longitudinal observational study

**DOI:** 10.3389/fpsyg.2025.1295848

**Published:** 2025-04-16

**Authors:** Stephanie Straub, Markus A. Hobert, Kirsten Emmert, Clint Hansen, Morad Elshehabi, Ulrike Suenkel, Isabel Wurster, Benjamin Roeben, Milan Zimmermann, Andreas J. Fallgatter, Daniela Berg, Walter Maetzler

**Affiliations:** ^1^Department of Neurodegeneration, Hertie Institute for Clinical Brain Research, University of Tübingen, Tübingen, Germany; ^2^German Center for Neurodegenerative Diseases (DZNE), Tübingen, Germany; ^3^Department of Neurology, University Hospital Schleswig-Holstein, Campus Kiel and Kiel University, Kiel, Germany; ^4^Department of Psychiatry and Psychotherapy, University Hospital Tübingen, Tübingen, Germany; ^5^German Center for Mental Health (DZPG), Partner Site, Tübingen, Germany

**Keywords:** unipolar depression, gait speed, neurodegenerative disorders, functional decline, cognition

## Abstract

**Introduction:**

Unipolar depression is a frequent psychiatric disorder and associated with cognitive and motor deficits. It is also a prodromal or risk marker for various neurodegenerative diseases. Little research has been done on the extent to which motor and cognitive deficits persist in remitted depressive episode, and to what extent these deficits can be progressive.

**Methods:**

A total of 401 healthy subjects of the Tübingen TREND study aged between 50 and 80 years were examined over 6 years at intervals of 2 years. Of those, 81 had a positive history of unipolar depression (Dep+) and 320 had not (Dep-). We ruled out currently depressive participants. Mini-Mental-State Examination (MMSE), Consortium to Establish a Registry for Alzheimer’s disease (CERAD), the motor part of the Movement disorder society-revised version of the Unified Parkinson disease rating scale (MDS-UPDRS III), gait speed, and dual tasking parameters were compared between groups.

**Results:**

Dep+ had lower gait velocity than Dep-. No significant change in parameters was found over the observed time.

**Discussion:**

Reduced gait speed in Dep+ may have potential as a diagnostic and prognostic marker for Dep- if confirmed in further studies. The lack of progression of all studied parameters over 6 years specifically in Dep+ argues against a relevant potential of this approach to define prodromal neurodegenerative cohorts by history of depression.

## Introduction

With a lifetime prevalence of 16%–20%, unipolar depressive disorder is one of the most common psychiatric conditions worldwide (Kessler et al., 2003; Jacobi et al., 2014; Maske et al., 2016). In 85%–94% of the cases it is associated with changes in cognitive performance, such as reduction of attention, executive function, and memory. These symptoms may persist beyond remission (Rovner et al., 1991; Doumas et al., 2012; Metzger et al., 2016). Unipolar depression is also associated with a 2–4-fold increased risk of developing (neurodegenerative) dementias and/or may be a prodromal symptom thereof (Byers and Yaffe, 2011; Kessing, 2012; Bennett and Thomas, 2014a). In addition, unipolar depressive disorder is associated with changes in motor performance, especially with a reduction of gait speed (Sloman et al., 1982; Metzger et al., 2016; Serrano-Checa et al., 2020; Hobert et al., 2023), and a prodromal marker of neurodegenerative diseases associated with motor dysfunction, particularly Parkinson disease (PD) (Lerche et al., 2014; Schrag et al., 2015; Del Din et al., 2019; Bareeqa et al., 2022; Lempriere, 2022).

However, to the best of our knowledge, whether depressive episodes lead to prolonged reductions in motor, especially gait speed, or cognitive performance beyond the acute depressive episode, has not been well investigated. This is particularly interesting as reduced gait speed in older adults is associated, among others, with increased risk of dementia, reduced independence of daily living (den Ouden et al., 2011; Artaud et al., 2015; Sanders et al., 2017; Binotto et al., 2018; Kamiya et al., 2018), increased hospitalization rate and increased mortality (Dumurgier et al., 2009; den Ouden et al., 2011; Studenski et al., 2011; Artaud et al., 2015). The aim of this 6 years longitudinal observational study was therefore to investigate various motor and cognitive parameters between participants with (Dep+) and without depressive episodes in their history (Dep-) over multiple assessments.

## Materials and methods

### Longitudinal TREND study and definition of the dataset used for the actual analysis

This is a sub-analysis from the still ongoing prospective longitudinal observational TREND study (*Tübinger Erhebung von Risikofaktoren zur Erkennung von Neurodegeneration*), conducted in the South of Germany. Approval for the study was obtained from the Ethics Committee of the Medical Faculty of Tübingen in 2009 (protocol number 90/2009BO2). A total of 1201 participants aged between 50 and 80 years were recruited from 2009 onwards and are re-assessed every 2 years (the study is still ongoing). Inclusion criteria were either the presence of hyposmia, rapid eye movement behavior disorder, and/or history of depressive episodes. In addition, healthy volunteers without any of these symptoms were included (Berg et al., 2015; Skorvanek et al., 2017). Exclusion criteria were relevant (other) neurological and psychiatric diseases, cognitive deficits, immobility (all as judged by the investigator), and taking neuroleptics, anticonvulsants, and benzodiazepines. Details of the study assessment protocol are available in Hobert et al. (2011).

**FIGURE 1:**
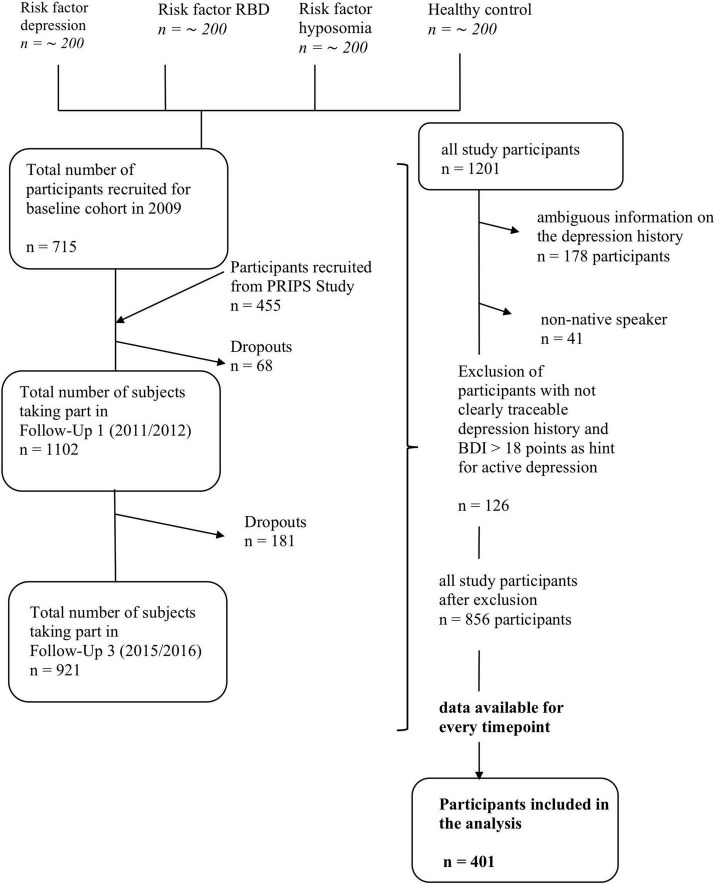
Flow chart for TREND participants included in this analysis. The left-hand side of the diagram shows the general process of the TREND study with corresponding fluctuations in the number of test subjects (drop-outs, further inclusions). The right-hand side shows the process of the analysis carried out with the exclusion of test subjects prior to the evaluation. RBD, Rapid Eye Movement Sleep behavior disorder; PRIPS Study, screening battery for subjects at risk for Parkinson’s disease.

In this analysis, data from visits 1–4 of 401 healthy participants were included. To rule out the effect of currently present unipolar depression, subjects with > 17 points on the Beck Depression Inventory I (BDI-I) were excluded from the analysis (group 2 and 3, *n* = 126 subjects). Details are presented in Figure 1. Of those, 81 were Dep+ (positive history of depression) and 320 Dep- (negative history of depression). A person was included in the Dep+ cohort if they indicated in a self-reported questionnaire, according to the model of the Federal Health Survey (Jacobi et al., 2002), and approved in a personal interview, that they suffered from physician-confirmed unipolar depression over the life course. Fourth, due to the design of the research question and the study, the extent to which the criteria for manifest depression were present could not be verified. Dep- was defined as answering the question in the negative. We excluded datasets of TREND participants whose information regarding their unipolar depression history was ambiguous or if no medical examination had taken place (group 4, *n* = 178 subjects). Non-native speakers were also excluded to prevent comprehension problems due to language (*n* = 41).

### Extracted parameters

The following parameters were extracted from the TREND database: BDI-I (Arnau et al., 2001) Mini-Mental-State Examination (MMSE) (Folstein et al., 1975; Morris et al., 1989), Consortium to Establish a Registry for Alzheimer’s disease (CERAD) test battery (Thalmann et al., 1997), motor part of the Movement disorder society-revised version of the Unified Parkinson disease rating scale (MDS-UPDRS III) (Goetz et al., 2008), fast gait speed and checking boxes speed from a single and dual tasking assessment, where participants walk 20 m on level ground in a corridor of at least 4 m width with the instruction to walk as fast as possible (Doumas et al., 2012; Metzger et al., 2016). Amount of sport activities was assessed with the German National Health Interview and Examination Survey 1999 (Mensink, 1999). Physical activity was defined by the type of sport and frequency (< 0.5 h/week, 0.5–1 h/week, > 1–2 h/week, > 2–4 h/week, and > 4 h/week). Deta**i**ls are provided in Table 1.

**TABLE 1:** Demographic data of participants with (Dep+) and without history of unipolar depression (Dep-).

Parameters	Dep-	Dep+	*P*-value	Combined
Participants (%)	320 (79.8)	81 (20.2)	–	401
Female (N) (%)	149 (46.6)	44 (54.3)	0.21	193 (48.1)
Age (y)	63.6	62.3	0.15	63.3
Education (y)	14.7	15.0	0.69	14.8
BDI-I (0–30)	4.4 ± 3.0	7.7 ± 3.4	< 0.001	5.1
**Activity levels**				
No activity	65 (23.3)	17 (19.8)	–	82
0.5 to 1 h per week	86 (30.8)	21 (24.4)	–	107
1 to 2 h per week	88 (31.5)	30 (34.9)	–	118
2 to 4 h per week	17 (6.1)	8 (9.3)	–	25
More than 4 h per week	23 (8.2)	10 (11.6)	0.5	33

Presented are mean and standard deviation values of age, and part of total (percentage). *P*-values of age, years of education and BDI I were calculated with the Mann-Whitney U test, for gender with the chi-square test. BDI-I, depression inventory, version 1. Difference in activity level was measures by chi-square test. The *P*-value in the last line takes into account the distribution of all activity levels between the groups.

### Statistical analysis

SPSS Statistics Version 24.0.0.0 (IBM) was used for statistical analyses. Distributions of age and years of education were compared between groups using the Mann-Whitney-U test, and distribution of gender using the chi-square test. Longitudinal analyses were performed using analysis of variance with repeated measures (rmANOVA, Park et al., 2009), as covariates we defined the BDI-I. The mean change in performance over time was calculated with the within-subjects effect. It depicts the influence of time on the parameters independent of the group assignment. Differences in the parameters between the two groups Dep+ and Dep-, independent of time, were calculated with the between-subjects effect (Brambor et al., 2006). Univariate rmANOVAs were calculated for the following parameters: BDI-I, MMSE, CERAD, MDS-UPDRS III, gait speed, and dual task costs (DTC). DTC were calculated based on Hobert et al. (2011). Significance level was set at 0.05. Bonferroni correction was applied for variance analysis with repeated measures (six tests, corrected values indicated).

## Results

**FIGURE 2:**
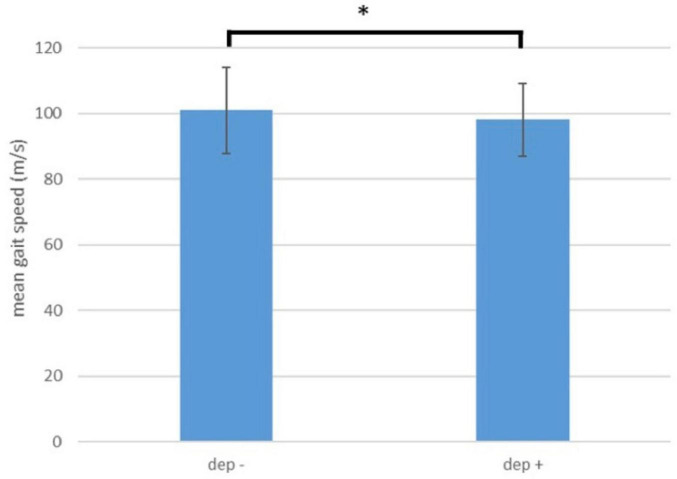
Comparison of gait speed between participants with (Dep+) and without history of depression.

Dep+ participants were not significantly different from Dep- participants in terms of age, gender and years of education, but Dep+ participants had higher BDI scores at baseline. Details are presented in Table 1. Gait speed was lower in Dep+ participants, compared to Dep- (mean for all time points 98 ± 11 m/min versus 101 ± 13 m/min, *p* = 0.048; Table 2). Over the observation period of 6 years, Dep+ and Dep- groups did not show a significant within-subjects effect in any of the rmANOVAs (MMSE, CERAD, MDS-UPDRS III, gait speed and DTC). Details, including *p*-values, F values and effect sizes are shown in Figure 2 and Table 2. Dep- did not show higher level of physical activity compared to Dep + (*p* = 0.5; Figure 1) based on the hours of exercise per week reported.

**TABLE 2:** Comparison of outcome parameters between participants with (Dep+) and without history of unipolar depression (Dep–).

Parameters	Dep-	Dep+	Between subject effect			Within subject effect		
			*P*-value	F value	Effect size	*P*-value	F value	Effect size
Mean gait speed (m/min)	101 ± 13	98 ± 11	0.048*	3.40	0.09	0.36	1.35	0.00
MDS UPDRS III (0–132)	1.8 ± 0.3	1.8 ± 0.3	0.11	1.92	0.01	0.57	0.45	0.00
CERAD (0–100)	85.4 ± 7.1	84.4 ± 8.0	0.65	1.26	0.00	0.49	0.62	0.01
MMST (0–30)	28.6 ± 0.9	28.6 ± 0.9	0.83	0.00	0.00	0.25	1.36	0.00
DTC (%)	+1.0 ± 0.5	+1.0 ± 0.5	0.65	0.34	0.00	0.52	0.64	0.00

Mean values are presented for all timepoints. CERAD, Consortium to Establish a Registry for Alzheimer’s disease; Dep-, negative history of unipolar depression; Dep+, positive history of unipolar depression; DTC, dual task cost for checking boxes during walking; MDS-UPDRS III, Movement Disorder Society Unified Parkinson disease rating scale III; MMST, Mini-Mental-State Examination.

*p-value < 0.05.

## Discussion

To the best of our knowledge, this is the first longitudinal observational case-control study that investigated motor and cognitive deficits after unipolar depressive episodes while considering relevant confounding factors. We found a significantly lower gait speed in participants with a positive history of unipolar depression over an observation period of 6 years. Our study adds relevant aspects to results of earlier studies showing a decrease in gait speed in individuals with unipolar depression (Everson-Rose et al., 2005; Michalak et al., 2009; Brandler et al., 2012). In more detail, our results indicate that the slowing of gait persists even after the persons affected recover from the depressive episode.

What could be the reasons for this phenomenon? One option is a persistent reduction of internal motivation and agility. This would be, at least indirectly, in line with a study showing that severity of unipolar depressive episode correlates negatively with gait speed (Brandler et al., 2012). However, as the BDI-I (which was indeed significantly higher in Dep+ than in Dep-) was considered as a covariate in our calculations, this explanation probably falls short, and it makes sense to look at other potential explanatory mechanisms. Another option could be a decrease of physical resources due to reduced activity in the context of, and after depression. Such an association has already been demonstrated (Penninx et al., 1999; Geerlings et al., 2001; Hybels et al., 2009), even in remission (Hiles et al., 2017; Schuch et al., 2017). However, as Dep+ and Dep- showed comparable physical activity levels, this option also does not seem to be a sound explanation for the observed differences. Eventually, it is also conceivable that people who have remitted from an unipolar depression walk more slowly due to a more relaxed or hesitant attitude to life, and thus slower gait speed in these people does not necessarily reflect a disease-associated deficit but rather a constitutional factor (Kermott et al., 2019; Laird et al., 2019). It could also be that people with slow gait speed are more prone to depressive episodes, which has to be investigated in future studies. Another noteworthy point is that we compared the fastest possible gait speed (capacity measure). It would be interesting to know whether Dep+ also have a slower gait speed in everyday life (performance measure). This aspect could be useful for the transfer to everyday functionality (Maetzler et al., 2021).

Interestingly, none of the motor and cognitive parameters investigated in this study deteriorated over the observation period of 6 years, particularly not in the Dep+ cohort. It has been suggested that subsyndromal depressive symptoms and slow gait are part of a geriatric syndrome that also includes impaired executive function (Albert, 2019; Patience et al., 2019). In addition, unipolar depression is associated with a 2–4-fold increased risk of dementia (Kessing, 2012; Bennett and Thomas, 2014b), which, in turn, is associated with reduced gait speed even in the prodromal phases (Metzger et al., 2016; Grande et al., 2019; Windham et al., 2022). Therefore, decline in gait speed in our cohort could have been interpreted as a “prodromal sign of cognitive impairment” although confirmation by clinical (dementia) diagnosis would have been helpful for validation with this respect. Moreover, unipolar depression is also associated with future development of PD (Schrag et al., 2015), and PD patients may have reduced gait speed over the last years before clinical diagnosis is possible (Del Din et al., 2019). As we did not find any evidence of a cognitive and motor deterioration over the observation period of 6 years in our groups, it remains unclear whether our groups were too small or the observation period too short to show significant changes over time. Another point is that the subject age may have been too low to reliably detect possible incipient neurodegenerative processes.

This study faces some limitations. First, although the TREND study overlooks a relatively large cohort of older adults, results may apply mainly for areas where the cohort was recruited (here in Central Europe). Second, two thirds of the original dataset could not be included in the analysis, due to the definition and completeness of the data available, so there remains some risk of bias due to the selection of datasets Third, we did not collect information about our participants before the depressive episode occurred that could potentially explain lower gait speed, and therefore cannot exclude the possibility that lowered gait speed in the Dep+ cohort is a constitutional factor. Moreover, it is also possible that the factors “slow gait speed” and “unipolar depressive episode” are only indirectly associated with each other. It therefore seems reasonable to confirm or reject the results we have presented here in another longitudinally observed cohorts.

In conclusion, we found in older adults that a history of unipolar depression in remitted persons is related to reduced gait speed. The extent to which constitutional and learned mechanisms play a role here cannot be determined in the end and needs to be determined in future studies. A steady decrease in function indicative of a neurodegenerative process could not be shown.

## Data Availability

The original contributions presented in this study are included in this article/supplementary material, further inquiries can be directed to the corresponding authors.
